# The COVID-19 outbreak increases maternal stress during pregnancy, but not the risk for postpartum depression

**DOI:** 10.1007/s00737-021-01104-9

**Published:** 2021-04-08

**Authors:** Myrthe G. B. M. Boekhorst, Lotte Muskens, Lianne P. Hulsbosch, Katrijn Van Deun, Veerle Bergink, Victor J. M. Pop, Marion I. van den Heuvel

**Affiliations:** 1 Department of Medical and Clinical Psychology, Tilburg University, Warandelaan 2, 5037 AB Tilburg, The Netherlands; 2 Department of Cognitive Neuropsychology, Tilburg University, Tilburg, The Netherlands; 3 Department of Methodology & Statistics, Tilburg University, Tilburg, The Netherlands; 4 Departments of Psychiatry and Obstetrics, Icahn School of Medicine At Mount Sinai, New York, NY USA

**Keywords:** COVID-19 pandemic, Pregnancy-specific stress, Perinatal, Depression

## Abstract

The COVID-19 pandemic affects society and may especially have an impact on mental health of vulnerable groups, such as perinatal women. This prospective cohort study of 669 participating women in the Netherlands compared perinatal symptoms of depression and stress during and before the pandemic. After a pilot in 2018, recruitment started on 7 January 2019. Up until 1 March 2020 (before the pandemic), 401 women completed questionnaires during pregnancy, of whom 250 also completed postpartum assessment. During the pandemic, 268 women filled out at least one questionnaire during pregnancy and 59 postpartum (1 March–14 May 2020). Pregnancy-specific stress increased significantly in women during the pandemic. We found no increase in depressive symptoms during pregnancy nor an increase in incidence of high levels of postpartum depressive symptoms during the pandemic. Clinicians should be aware of the potential for increased stress in pregnant women during the pandemic.

## Introduction

The outbreak of the COVID-19 pandemic and subsequently the lockdown has had a substantial impact on society, especially for vulnerable groups in the population such as pregnant women. Pregnancy and the postpartum period are already vulnerable periods of time, which can co-occur with heightened levels of distress in many women (Woody et al. 2017). Moreover, this pandemic has led to substantial changes in obstetric care; for example, the frequency of face-to-face consultations decreased during pregnancy (Coxon et al. 2020). Pregnant women had to deal with the anxiety of infection, along with many other uncertainties such as the concern that their partner may not be present at delivery. On top of that, there is very limited knowledge about the susceptibility or altered disease course for COVID-19 during pregnancy, and what the possible effect might be for the unborn child. The social distancing guidelines and travel restrictions may also have resulted in increased social isolation (Lebel et al. 2020; Usher et al. 2020). Consequently, it might have been difficult to bond with other pregnant women, also because of cancelled perinatal classes. Furthermore, postpartum women may not have been able to celebrate the birth of the baby with friends and family and had to deal with stress and exhaustion without assistance of friends, family, or professional caregivers due to the COVID-19 guidelines. Additionally, much time was spent at home during the pandemic, which in some cases has led to strained relationships with the partner during the pandemic (Lebel et al. 2020). In a Canadian study, it was found that 18% of the women lost their job due to the COVID-19-pandemic (Lebel et al. 2020), which could have caused financial uncertainties. Together, these COVID-19-related changes have the potential to increase fear and worries in pregnant women (Ravaldi et al. 2020), and impact perinatal mental health.

Understandably, research on the impact of the COVID-19 pandemic on perinatal women’s mental health is still very sparse. The first reports on this topic show higher prevalence of perinatal depression and anxiety during the COVID-19 pandemic, as compared to norm data before the pandemic, both in pregnant and postpartum women (Ceulemans et al. 2020; Lebel et al. 2020). In another study, the incidence of high depression and high anxiety scores were higher in pregnant and postpartum women during pregnancy as compared to pre-pandemic scores that women retrospectively recalled (Davenport et al. 2020). During the pandemic, the incidence of maternal depression and anxiety was also found to be higher in mothers of children aged 0 to 8 years (Cameron et al. 2020). These studies show that perinatal women may be especially vulnerable to psychological distress during the COVID-19 pandemic. Nonetheless, these studies have several major methodological shortcomings, particularly by using cross-sectional data, retrospective measurements, and/or comparing pandemic data to norm data (no matching control group). On the other hand, Pariente et al. (2020) found that postpartum women during the COVID-19 pandemic had a lower risk for high depression scores shortly after giving birth compared to a control group of women who gave birth a few years prior to the pandemic. In addition, Silverman et al. (2020) found an improvement in depressive symptoms during pregnancy in women with low socioeconomic status, after the implementation of social restrictions compared to the early stages of the COVID-19 pandemic. While these studies provide important information, studies that are able to compare pregnant women during the pandemic with a matching control group of women that were pregnant *right before* the pandemic are necessary to make better inferences about the mental health effects of the pandemic on the pregnant population.

The current prospective cohort study from the Netherlands, the Brabant Study (Meems et al. 2020), provides the unique opportunity to fill this gap. The Brabant Study is one of very few studies worldwide for which inclusion continued during the COVID-19 pandemic. The recruitment started in 2019 and continued during the pandemic, as well as during the 3-month-long strict nationwide lockdown (March–May 2020). Moreover, Brabant is in the south of the Netherlands, which proved to be one of the pandemic epicenters in Europe. Consequently, the current study provides a unique opportunity to compare symptoms of depression and stress in the perinatal period during and right before the COVID-19 pandemic.

## Method

### Participants and procedure

**Fig. 1:**
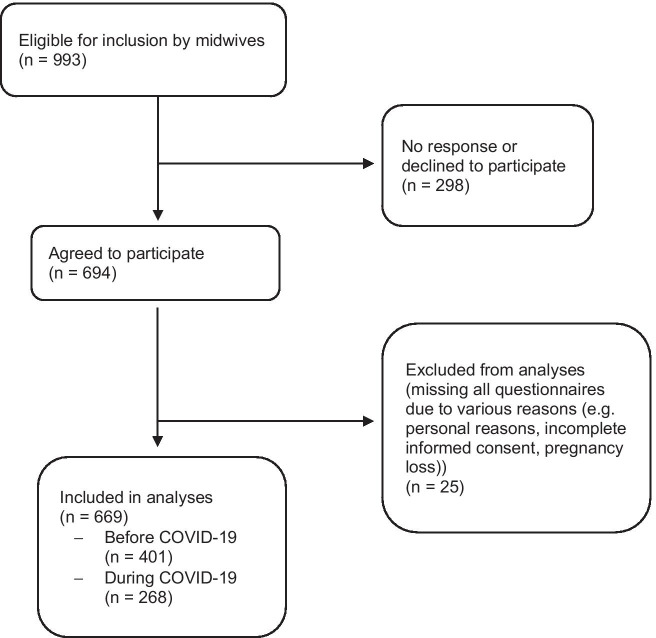
Flowchart of participant inclusion

The current study is part of a longitudinal prospective cohort study (the Brabant Study) (Meems et al. 2020) among pregnant women who are followed from 12 weeks pregnancy until 10 weeks postpartum. Eligible pregnant women were recruited by community midwife practices and hospitals in Brabant, the Netherlands. After a pilot in 2018 (started 13 May 2018), recruitment started on 7 January 2019 and is still ongoing. Details on the design of the Brabant Study are described elsewhere (Meems et al. 2020). In short, Dutch pregnant women (18 + years) who had their first antenatal visit before 14 weeks of gestation were eligible for participation. Exclusion criteria were as follows: multiple pregnancy, known endocrine disorder before pregnancy (other than thyroid function problems), diabetes type I, rheumatoid arthritis, severe psychiatric disease (schizophrenia, borderline personality disorder, or bipolar disorder), HIV, drug or alcohol addiction problems or any other disease resulting in treatment with drugs that are potentially adverse for the fetus and need careful follow-up during pregnancy. Moreover, women must have access to the internet. As reported by community midwives, 70% of the women who met inclusion criteria were willing to participate. This reflects 993 women to be eligible to participate up until mid-May 2020, of which 694 indicated that they were willing to participate. Women were included in the analyses if they had completed at least one questionnaire during pregnancy (12, 20, or 28 weeks of pregnancy). Of these 694 women, 25 (3.6%) failed to complete any of the three questionnaires during pregnancy due to various reasons (e.g., personal reasons, incomplete informed consent, or pregnancy loss) (see Fig. 1 for flowchart of participant inclusion).

Participating women completed online questionnaires during all three trimesters of pregnancy and 8 to 10 weeks postpartum. Up until 1 March 2020, before the COVID-19 pandemic started in the Netherlands, 401 women completed questionnaires during pregnancy (trimester 1: *N* = 393; trimester 2: *N* = 350; trimester 3: *N* = 350), of whom 250 also completed postpartum assessment. During the pandemic, from 1 March 2020 to 14 May 2020, 268 women filled out at least one questionnaire during pregnancy (trimester 1: *N* = 265; trimester 2: *N* = 203; trimester 3: *N* = 110), and 59 completed postpartum assessment. This resulted in data of 669 participants to be analyzed in the current study. Because the pandemic period was defined as the 3-month nationwide strict lockdown, this study period ended before all women had completed assessment in the third trimester of pregnancy or postpartum. During pregnancy 436 completed all questionnaires, 131 completed two questionnaires and 102 completed one questionnaire.

These participating women had a mean age of 30.83 (SD = 3.66), 66.6% was highly educated (Bachelor’s degree or higher), and 97.4% had a Dutch ethnic background. The majority of women were employed (94.8%), of which only 4 (0.6%) indicated they would not return to work after maternity leave. Of the 669 participating women, 98.8% had a partner, of which 98.9 were in a heterosexual relationship. The majority of the partners were employed (99.4%). Furthermore, 316 (48.2%) women were primiparous, 164 (24.8%) women had a previous miscarriage or abortion, 48 (7.3%) women had an unplanned pregnancy, and 77 (11.5%) women had a previous diagnosis of depression. Table 1 shows the demographic characteristics of the women who were pregnant before and during the COVID-19 pandemic. The demographic characteristics between the pandemic and pre-pandemic group were similar with regard to age, education, employment, marital status, parity, unplanned pregnancy, previous miscarriage, and previous diagnosis of depression.

**Table 1:** Demographic characteristics of women who were pregnant before and during the COVID-19 pandemic (*N* = 669)

Pregnancy (*N* = 669)	Pre-pandemic group (*N* = 401)				Pandemic group (*N* = 268)			
Demographics	*N*	%	Mean (SD)	Range	*N*	%	Mean (SD)	Range
Age	395		30.88 (3.67)	21–41	265		30.75 (3.64)	19–45
High education	255	64.6			184	69.7		
Employment	372	94.7			252	95.1		
Having a Partner	380	98.4			260	99.2		
Primiparous	177	45.4			139	52.5		
Unplanned pregnancy	24	6.1			24	9.1		
Previous miscarriage	96	24.3			68	25.7		
Previous diagnosis of depression	36	11.6			41	15.5		
BMI	391		24.01	17–42	265		24.08	18–37

The pandemic group completed at least one questionnaire between 1 March 2020 and 14 May 2020. High Education, Bachelor’s degree or higher; SD, standard deviation

The study was approved by Medical Ethics Committee at the Máxima Medical Centre Veldhoven (L64091.015.17). All participants provided written informed consent.

### Measures

#### Depressive symptoms

The 10-item Edinburgh (Postnatal) Depression Scale (E(P)DS) was used to measure depressive symptoms during pregnancy and postpartum (Cox et al. 1987). The E(P)DS is a frequently used and widely applicable instrument for perinatal use (O’Connor et al. 2016). Items were rated on a 4-point Likert-type scale. Total scores range between 0 and 30, with higher total scores indicating higher levels of depressive symptoms. A score higher than 12 was used to identify the women at risk for high levels of postpartum depressive symptoms (Cox et al. 1987). The Cronbach’s alpha of the E(P)DS varied between α = 0.85 and α = 0.86 in the current study.

#### Pregnancy-specific stress

We assessed pregnancy-specific stress using the 10-item adapted version of the negative affect subscale of the Tilburg Pregnancy Distress Scale (TPDS-NA). The scale assesses worries during pregnancy about fetal health, childbirth, and delivery (Boekhorst et al. 2020). Examples of items are “I worry about the pregnancy” and “I get very tense hearing stories about deliveries.” Items were rated on a 4-point Likert-type scale (0 = very often, 1 = fairly often, 2 = now and then, 3 = rarely or never). Total scores range from 0 to 30, with higher total scores indicating higher levels of pregnancy-specific stress. The TPDS showed good psychometric properties regarding internal consistency, test–retest reliability, hypotheses testing, and concurrent validity (Boekhorst et al. 2020) and has been reviewed as excellent in terms of its internal consistency and structural validity (Evans et al. 2015). Since its development, the TPDS has been translated into various languages such as, among others, English, Portuguese, Turkish, Spanish, Mandarin, and Japanese. The TPDS-NA has been shown to correlate significantly with the E(P)DS at all trimesters of pregnancy (range *r* = 0.50–0.54, all *p* < 0.001) (Boekhorst et al. 2020) and with the Generalized Anxiety Disorder–7 (*r* = 0.52, *p* < 0.001) (Pop et al. 2011). The Cronbach’s alpha ranged from α = 0.80 to α = 0.86 for the TPDS-NA in the current study.

### Statistical analyses

Mixed models statistics were used to analyze the possible effect of the pandemic on the individual trajectory of depression and stress symptoms over time (different trimesters of pregnancy). We selected covariates based on theory (age, education, parity, previous depression, previous miscarriage, unplanned pregnancy, and employment) (for review see Biaggi et al. 2016). For mixed model analyses, all cases can be included, including those that do not have measurements for every point in time (Bagiella et al. 2000). Therefore, all participants that completed at least one assessment during pregnancy were included in the analyses. As an assistance to the interpretation of results, the significant coefficients in terms of percentage change in symptoms per unit change [formula: (expβ-1)*100] were reported. Next, logistic regression analysis was used to examine whether perinatal pandemic women were more likely (OR, 95%CI) to develop high levels of postpartum depressive symptoms than pre-pandemic women, using the predefined postpartum cut-off (> 12) for the E(P)DS.

## Results

The Pearson r correlations between the E(P)DS and the TPDS-NA were *r* = 0.49 at trimester 1, *r* = 0.50 at trimester 2, and *r* = 0.56 at trimester 3 (all *p* < 0.001).

For the pre-pandemic group, the median score on the E(P)DS was 4 (IQR = 6) in the first trimester, 4 (IQR = 5) in the second trimester, and 4 (IQR = 7) in the third trimester. For the pandemic group, the median score on the E(P)DS was 4 (IQR = 5) in the first trimester, 4 (IQR = 6) in the second trimester, and 5.5 (IQR = 6) in the third trimester. With regard to the TPDS-NA, the pre-pandemic group had a median score of 4 (IQR = 4) in the first trimester, 4 (IQR = 5) in the second trimester, and 3 (IQR = 6) in the third trimester. The pandemic group had a median TPDS-NA score of 5 (IQR = 5) in the first trimester, 4 (IQR = 6) in the second trimester, and 4 (IQR = 7) in the third trimester.

Results of mixed model analyses showed that for the E(P)DS-model, the main effect of the pandemic was not a significant predictor of depressive symptoms throughout pregnancy (*β* = − 0.03, SE = 0.32, *t* = − 0.09, *p* = 0.925). The results of the current study showed that there was a slight increase in depressive symptoms from trimester 1 to trimester 3, but this effect of time was not significant (F (2, 881.14) = 1.21, *p* = 0.300).

**Fig. 2:**
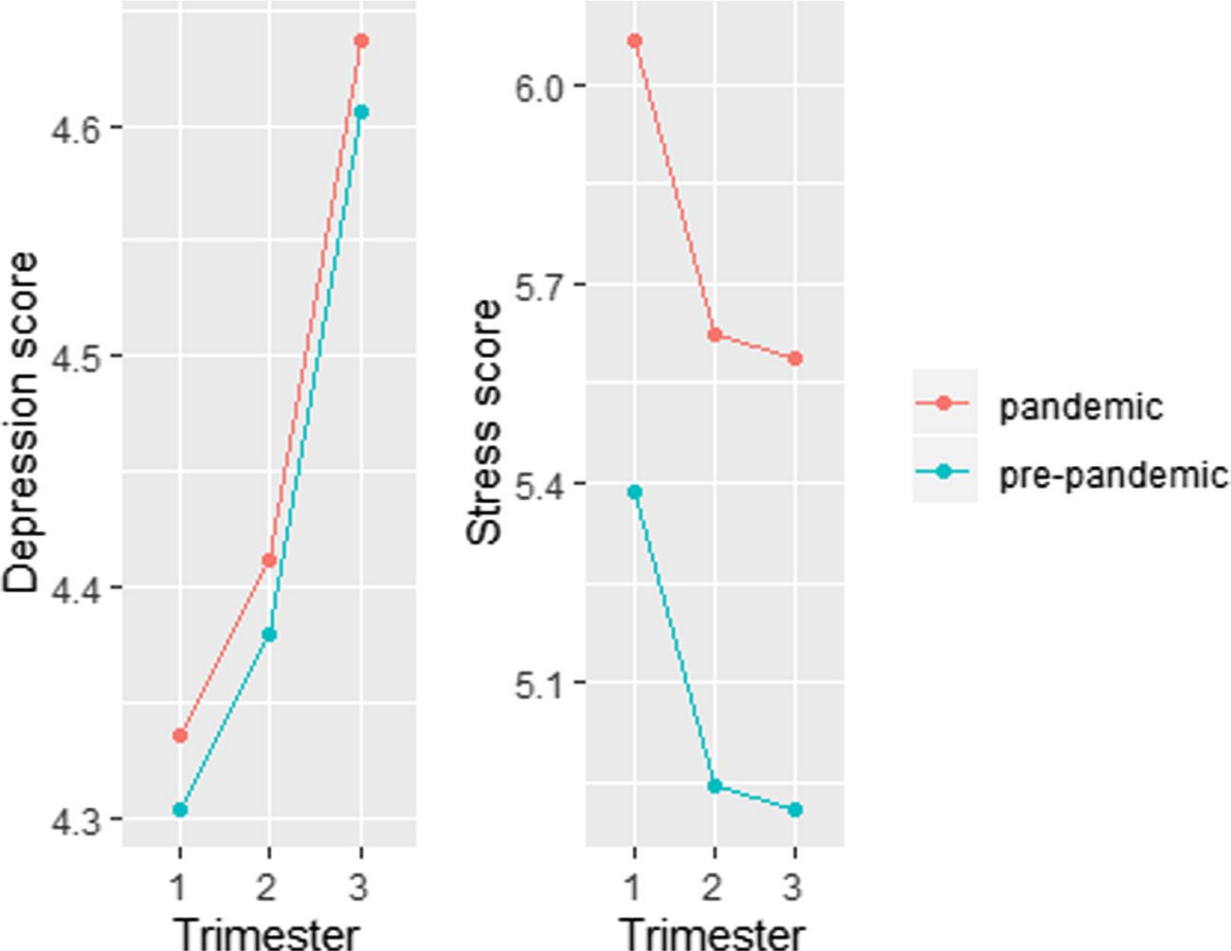
Mean depression and stress symptom scores during gestation for women who were pregnant pre-pandemic (blue line) compared to those pregnant during the pandemic (red line). There were no differences in depression scores but women who were pregnant during the pandemic had significantly higher stress scores compared to non-pandemic women. The axes have a different range for the EPDS and TPDS; the pandemic group completed at least one questionnaire between 1 March 2020 and 14 May 2020. Depression score pre-pandemic: *N* = 393 at T1, *N* = 350 at T2, and *N* = 350 at T3; depression score pandemic: *N* = 265 at T1, *N* = 203 at T2, and *N* = 110 at T3. Stress score pre-pandemic: *N* = 312 at T1, *N* = 313 at T2, and *N* = 330 at T3; Stress score pandemic: *N* = 265 at T1, *N* = 203 at T2, and *N* = 111 at T3

However, the TPDS-NA-model showed a main effect of pandemic (*β* = − 0.69, SE = 0.32, *t* = − 2.13, *p* = 0.034) on pregnancy-specific stress symptoms. The beta coefficient can be explained as the percentage change in stress per unit change in the pandemic group, corresponding to 49.7% higher stress scores in the pandemic group. The effect of time on stress during pregnancy was significant (F (2, 870.46) = 5.87, *p* = 0.003), showing a decrease over time. Compared with trimester 3, there was a significant difference in stress scores for trimester 1 (β = 0.48, SE = 0.17, *t* = 2.88, *p* = 0.004) but not trimester 2 (β = 0.04, SE = 0.17, *t* = 0.23, *p* = 0.824). Figure 2 provides a graphical overview of the results.

Seven percent of the pre-pandemic and 8.5% of the pandemic women had a score higher than 12 on the E(P)DS at 8–10 weeks postpartum, which may suggest high levels of postpartum depressive symptoms. Belonging to the pandemic group was not related to high levels of postpartum depressive symptoms (OR = 1.24; 95%CI: 0.44–3.50; *p* = 0.689).

## Discussion

Stress symptoms increased significantly in pregnancy during the COVID-19 outbreak. Interestingly, we did not see a rise in depressive symptoms during pregnancy nor an increase in incidence in high levels of postpartum depressive symptoms during the pandemic. Although studies have shown that there is an association between prenatal symptoms of anxiety and postpartum depressive symptoms (Grigoriadis et al. 2019), the presence of COVID-19-related worries and anxiety during pregnancy might be unrelated to postpartum depression. It could be that after childbirth has passed, which is a potential stressful event during the COVID-19 pandemic, COVID-19-related anxiety decreases, especially when the newborn is healthy.

Our findings could be of clinical importance, especially if replicated in populations with lower education and single-parent households, as well as in other countries. Our results indicate that many pregnant women may suffer from stress during the COVID-19 outbreak, but these symptoms may not be detected during routine screening. The E(P)DS is a widely used screening instrument to assess depressive symptoms during pregnancy (O’Connor et al. 2016), but does not measure pregnancy- and delivery-related worries and anxiety. While we fully support the E(P)DS as screening instrument during pregnancy to detect increased depressive symptoms, the use of the E(P)DS may not be sensitive enough to detect COVID-19-induced stress in pregnant women and could lead to underestimation of the mental health burden. Ideally, clinicians could consider adding screening instruments for stress symptoms, especially during the COVID-19 outbreak. The TPDS-NA is appropriate for the assessment of pregnancy-specific worries and stress, but adjusted scales to assess COVID-19-related stress may be even more appropriate for this population (e.g., Taylor et al. 2020). In addition, future studies should assess COVID-19-related stress and worries specific to perinatal women.

Moreover, the complexity of appropriate and effective mental health treatment for mothers during the pandemic should be further examined in future studies, especially a comparison between effectiveness of face-to-face treatment and customized treatment to the pandemic (e.g., virtual). In their meta-synthesis, Shorey and Chan (2020) drew on experiences from past epidemics and pandemics, concluding a need for technology-based interventions and psychosocial interventions for mental health care in pregnant women. An example could be an online mindfulness intervention that has shown to reduce levels of distress (Spijkerman et al. 2016), but future studies should assess its effectiveness in pregnant women (Hulsbosch et al. 2020).

## Strengths and limitations

The current study has a number of strengths and limitations that should be mentioned. A key strength of this study is the longitudinal design of our cohort, which allowed us to measure symptoms of stress and depression during the course of pregnancy, as well as to compare symptoms before and during the pandemic. Nevertheless, the following limitations should also be considered. First, the sample that was assessed in the current study consisted solely of Dutch women. This may limit generalizability of results to other countries with different cultures, health care systems, and standard of living. Additionally, the participants were predominantly (66.6%) highly educated. This rate is higher compared to the general female population in the Netherlands with a similar age category, where approximately 48–56% was highly educated between 2019 and 2020 (Statistics the Netherlands 2020b). Furthermore, participants in the current more often had a partner compared to the general Dutch population. It has been shown that 8.8% of the children born in 2019 in the Netherlands were born in a single-parent household (Statistics the Netherlands 2020a). Therefore, generalization of our results could be restricted. Furthermore, even though the longitudinal design of the current study allowed us to assess the course of symptoms during pregnancy, the current design did not allow for an assessment of change in symptoms of depression and stress from the prenatal to postnatal period, nor a comparison in this change between the pandemic and pre-pandemic group.

As the pandemic progresses, future studies should be able to address the longitudinal effects of the COVID-19 pandemic on depressive and stress symptoms (from pregnancy to postpartum and early parenthood). Another limitation of the study is the smaller sample size for the postpartum assessment of the pandemic group (*N* = 59) compared to the pre-pandemic group (*N* = 250), suggesting careful interpretation of postpartum results. Finally, we assessed high levels of depressive symptoms with a self-report instrument, the EPDS, and not with a diagnostic interview. However, based on their review, O’Connor et al. (2016) concluded that the E(P)DS is a frequently used and widely applicable screening instrument, and found that the sensitivity of the EPDS ranged between 0.67 and 1.00 and specificity was higher than 0.87 in the included studies.

## Conclusions

Our findings indicated that the COVID-19 pandemic induces worries in pregnant women in the Netherlands. Given that fetal exposure to stress can have detrimental effects on child brain development (Van den Bergh et al. 2017), we conclude that it is important for clinicians to be extra aware of pregnant women with increased levels of stress during the COVID-19 pandemic. It is of great importance that adequate mental health care and support is provided for mothers in need (Hermann et al. 2020).
